# Physical Activity, Screen Time, and Neurobehavioural Function at Age 7 Years: The Italian PHIME-NAC-II Cohort

**DOI:** 10.3390/children13101442

**Published:** 2026-10-21

**Authors:** Fabiano Barbiero, Valentina Rosolen, Marika Mariuz, Maria Parpinel, Luca Ronfani, Liza Vecchi Brumatti, Maura Bin, Agnese Zanin, Luigi Castriotta, Francesca Valent, Fabio Barbone

**Affiliations:** 1 UOC Unit of Occupational Medicine, Department of Medicine, Surgery and Health Sciences, University of Trieste, 34129 Trieste, Italy; 2 Central Directorate for Health, Social Policies and Disability, Friuli Venezia Giulia Region, 34121 Trieste, Italy; valentina.rosolen@regione.fvg.it (V.R.); marika.mariuz@regione.fvg.it (M.M.); 3 Department of Medicine (DMED), University of Udine, 33100 Udine, Italy; maria.parpinel@uniud.it; 4 Institute for Maternal and Child Health, IRCCS (Scientific Institute for Research, Hospitalization and Healthcare) ‘Burlo Garofolo’, 34137 Trieste, Italy; luca.ronfani@burlo.trieste.it (L.R.); liza.vecchibrumatti@burlo.trieste.it (L.V.B.); maura.bin@burlo.trieste.it (M.B.); 5 Residency Program in Hygiene and Preventive Medicine, Department of Diagnostics and Public Health, University of Verona, 37134 Verona, Italy; agnese.zanin@studenti.univr.it; 6 Institute of Hygiene and Evaluative Epidemiology, Friuli Centrale University Health Authority, 33100 Udine, Italy; luigi.castriotta@asufc.sanita.fvg.it; 7 Hygiene and Public Health, Friuli Centrale University Health Authority, 33100 Udine, Italy; francesca.valent@asufc.sanita.fvg.it; 8 Department of Medicine and Health Sciences (DiSMed), University of Trento, 38122 Trento, Italy

**Keywords:** physical activity, child behaviour checklist, Wechsler intelligence scale for children, cohort study, early childhood, interactive and passive screen time exposure

## Abstract

**Highlights:**

**What are the main findings?**

- Higher physical activity and lower passive screen exposure were associated with favorable behavioural health indicators.
- Greater passive screen time (television viewing) was associated with higher internalizing, externalizing, and total behavioural problems, whereas interactive screen time was not associated with behavioural or cognitive outcomes.

**What are the implications of the main findings?**

- Interventions should consider the local built environment and opportunities for accessible, structured physical activity when supporting healthy movement behaviours in children.
- Due to the worldwide prevalent child exposure to screens and initial signals of possible neurobehavioural effects, further, dedicated prospective research and monitoring on screen exposure and behavioural health indicators in school-aged children is recommended.

**Abstract:**

**Objective:** To investigate the cross-sectional associations of physical activity (PA) and interactive and passive screen-time (ST) exposure with behavioural and cognitive development at age 7 years within the PHIME-NAC-II prospective birth cohort. **Methods:** At age 7 years, 482 children from Trieste, North-eastern Italy underwent assessment of behavioural and emotional problems (CBCL) and general cognitive functioning (WISC-IV FSIQ). Parents reported PA (days/wk with ≥1 h of sports or active play), interactive ST exposure (hr/day playing video games, computers, tablets) and passive ST (television watching). Socio-demographic and lifestyle information was collected from gestation to age 7 years. Multiple linear regression models examined the associations between PA and ST variables and internalizing (IntP), externalizing (ExtP), total (TotP) behavioural problems and FSIQ. **Results:** PA showed quadratic associations consistent with a U-shaped pattern for IntP (B_PA_ = −0.77, *p* = 0.048; B_PA^2_ = 0.32, *p* = 0.029), and TotP (B_PA_ = −0.59, *p* = 0.100; B_PA^2_ = 0.30, *p* = 0.022), and predicted score minima ≈ 3.5–3.8 days/wk, while no clear association was detected with ExtP and FSIQ. Higher passive ST was associated with worse IntP, ExtP and TotP. Interactive ST was not associated with outcomes. **Conclusions:** engaging during school age in regular PA—with the lowest predicted behavioral problem scores observed around 3–4 days/week—was associated with lower problem scores, independent of passive screen-time exposure.

## 1. Introduction

During childhood the brain and behaviour are undergoing rapid maturation, and lifestyle factors such as physical activity (PA) may influence neurodevelopmental trajectories. In recent years an increasing body of evidence has linked higher levels of PA with more favorable behavioural and cognitive outcomes in children [1,2,3]. Engagement in regular PA during childhood is positively associated with neurocognitive development: evidence indicates that aerobic activity enhances executive functions, such as working memory, attention, inhibitory control and cognitive flexibility [4]. Beyond cognitive outcomes, PA has also been associated with better behavioural and emotional regulation in children, including lower levels of internalizing and externalizing problems [5]. In contrast, excessive sedentary behaviour—particularly recreational screen time (ST)—has been linked to poorer neurodevelopmental outcomes [6,7]. Higher recreational ST has also been consistently linked to greater behavioural and emotional difficulties, including poorer self-regulation and increased internalizing symptoms in children [8]. Moreover, higher sedentary time is associated with weaker language, attention, academic performance, and lower self-esteem [9], while prolonged ST use—a major contributor to sedentary behavior—is also related to reduced social interaction, emotional dysregulation, and greater internalizing problems such as anxiety and depression [10]. Neuroimaging evidence further indicates that heavy screen exposure may be associated with reduced white-matter integrity in tracts supporting language and literacy [11]. The World Health Organization’s (WHO) Guidelines on Physical Activity and Sedentary Behavior for children and adolescents (ages 5–17 years), the Italian National Institute of Health (Istituto Superiore di Sanità, ISS), and the Canadian 24-Hour Movement Guidelines for Children and Youth [12,13] recommend that children and adolescents aged 5–17 years accumulate an average of at least 60 min per day of moderate to vigorous PA, most of which should be aerobic. In addition, they emphasise that vigorous aerobic activities and activities that strengthen muscles and bones should be incorporated at least 3 days per week. WHO also notes that doing some PA is better than none, and that opportunities should be provided so all children and adolescents can participate in varieties of enjoyable and age-appropriate activities. Its guidelines also stress the importance of limiting sedentary behavior, particularly recreational ST, and reducing prolonged sitting, although no specific universal threshold is provided for older children and adolescents [13]. For younger children, WHO recommends no ST for those under 1 year and no more than 1 h per day for children aged 2–4 years [14]. In contrast, several national and international guidelines, such as the Canadian 24-Hour Movement Guidelines and earlier recommendations from the American Academy of Pediatrics, have suggested limiting recreational ST to approximately 2 h per day for school-aged children and adolescents [15].

Additionally, children and youth should obtain 8–11 h of sleep each night, depending on age, with consistent bedtimes and wake times.

Despite growing interest in how PA and ST exposure relate to children’s behavior and cognition, few studies have examined simultaneously these associations using standardised tests of behavioural and emotional problems and general intellectual ability such as the Child Behaviour Checklist (CBCL) [16], and the Wechsler Intelligence Scale for Children (WISC) Full-Scale IQ (FSIQ), respectively [17].

Furthermore, several existing studies focus on domain-specific cognitive functions such as executive function, attention, and memory, rather than global cognitive ability (e.g., FSIQ) or behavioural outcomes assessed through standardised checklists (e.g., CBCL) [18]. As a result, the combined use of validated measures capturing both global cognitive functioning and behavioural outcomes in relation to PA remains limited in the current literature. In addition, interactive and passive forms of screen exposure may have different relationships with child development, but these exposures are not consistently distinguished across studies. Despite these limitations, evidence concerning screen exposure and behavioural problems is relatively consistent, although the magnitude of the observed associations is generally small. A systematic review and meta-analysis of children aged 12 years or younger reported small but significant associations between greater screen-time duration and both externalizing and internalizing behavioural problems, while also highlighting substantial heterogeneity related to measurement and methodological characteristics [19]. More recent evidence focusing on 24 h movement behaviours also suggests that associations with behavioural outcomes may vary according to the specific movement behaviour considered and the way exposures and outcomes are operationalized. In a systematic review of children, Hu et al. [20] found more consistent evidence for associations between adherence to screen-time and sleep guidelines and externalizing problems, whereas evidence for internalizing problems was less clear. In contrast, evidence concerning the relationship between PA and behavioural outcomes remains more heterogeneous and appears to vary according to how PA is defined and measured. These differences in exposure assessment are important when comparing findings across studies and suggest that the present study should be considered a conceptual or analytic replication of previous observational research examining associations between PA, screen exposure, and behavioural outcomes, rather than a direct replication. Moreover, existing studies often do not account for key factors that may influence behaviour, cognition, and PA. Many potential confounders and relevant covariates—spanning prenatal, early-life, and contemporaneous environments, as well as broader biological and familial factors—are not consistently included, which may limit the comparability of findings across studies and the ability to assess the independent associations of PA and ST with developmental outcomes. A multivariable approach is therefore essential to clarify the independent associations of PA and ST with behavioural and cognitive functioning at this crucial developmental stage. Taken together, current evidence underscores the need for studies focusing specifically on children in early school age, integrating behavioural (CBCL) and cognitive (FSIQ) outcomes while adjusting for a broad set of relevant biological, environmental, and lifestyle covariates. Examining these associations within a prospective birth cohort may therefore provide additional evidence on the relationships between PA, different forms of screen exposure, and developmental outcomes at school age. Addressing these limitations is particularly important at age of 7 years, that represents a particularly pivotal stage of childhood, characterized by rapid advances in logical reasoning and self-regulated behaviour, increasing social and emotional competence, and greater engagement with structured educational environments [21].

Therefore, this study aims to investigate the independent associations of PA and screen exposure in mid-childhood with behavioural and emotional functioning as well as cognitive performance in 7-year-old children whose mothers were enrolled during pregnancy in the Northern Adriatic Cohort II (NAC-II), considering a broad set of relevant biological, environmental, and lifestyle predictors. The Italian NAC-II is part of the Mediterranean cohort involved in the ‘Public health impact of long-term, low-level, mixed element exposure in susceptible population strata’ (PHIME) project [22].

## 2. Materials and Methods

### 2.1. Study Population

The original base for this research included pregnant women attending routine ultrasound examinations at the IRCCS Burlo Garofolo in Trieste, Italy—the only maternity hospital in city, where more than 95% of births among city residents took place. They were invited to join the NAC-II prospective birth cohort, established within the PHIME project [20]. The present secondary analysis includes 482 children who participated in the follow-up at 7 years of age, completed the WISC-IV [21], and had a parent-reported CBCL completed on the same day. As no additional sampling procedure was undertaken, the analytical sample size was determined by participation in the cohort and the availability of both age-7-years assessments. Inclusion was not based on the children’s IQ or socioeconomic status: FSIQ was treated as a developmental outcome, whereas socioeconomic status was measured using the SES index and included as a covariate in the multivariable analyses. A detailed description of the cohort protocol has been published elsewhere [20,22].

### 2.2. Eligibility Criteria

Women were eligible for inclusion if they were of legal age, permanently residing in the Friuli-Venezia Giulia region for at least two years and had not been absent from the region for more than six weeks during pregnancy. Eligible women were approached immediately after the routine morphological ultrasound performed between the 20th and 22nd weeks of gestation and were informed about the aims of the study. Those who agreed to participate signed informed consent and completed a short preliminary questionnaire to screen for possible exclusion criteria and to provide basic family information.

### 2.3. Inclusion and Exclusion Criteria

Only singleton pregnancies were considered, and women with a history of drug abuse, major health problems or pregnancy complications that could potentially interfere with the normal neurological development of the child were excluded. Children with congenital malformations, severe perinatal complications or major health problems arising in the months following birth that could compromise neurological development were excluded. These exclusion criteria were adopted to minimize confounding factors associated with developmental delay.

### 2.4. Mothers’ Questionnaires

At recruitment, between the 20th and 32nd week of gestation, mothers who agreed to participate in the study completed a short preliminary questionnaire to screen for possible exclusion criteria and to provide basic family information. Furthermore, mothers were tested with the non-verbal intelligence test, Raven’s Progressive Matrices [23], to estimate maternal intelligence quotient (IQ). They were also given a detailed questionnaire to be returned one month postpartum, collecting information on demographic and socioeconomic characteristics, maternal and infant health during pregnancy, delivery and the first month of life, as well as lifestyle, dietary and alcohol habits throughout pregnancy. At age 7 years, a Supplementary Questionnaire was filled by the parents to detect changes in socio-demographic information and lifestyle habits of the child and family. Children’s height and weight were also measured by health professionals. Further details have been reported elsewhere [22,24].

### 2.5. Child Assessment at Age 7 Years

Behavioural functioning was evaluated using the CBCL, a validated questionnaire that measures parental perception of their child’s behaviour and emotions over the preceding six months. The CBCL is a widely used standardised instrument with strong psychometric properties, including high internal consistency (Cronbach’s α ranging from 0.88 to 0.93 for composite domains) and good test–retest reliability across population-based samples [16,25]. The CBCL comprises 118 items rated on a three-point Likert scale (0 = “not true”, 1 = “somewhat or sometimes true”, 2 = “very true or often true”), grouped into eight syndrome scales (anxious/depressed, withdrawn/depressed, somatic complaints, social problems, thought problems, attention problems, rule-breaking behaviour and aggressive behaviour).

These scales are then combined into two empirically derived composite domains: (i) the internalizing problems domain (IntP), a measure of emotional problems (sum of anxious/depressed, withdrawn/depressed and somatic complaints), and (ii) the externalizing problems domain (ExtP), a measure of behavioural problems (sum of rule-breaking and aggressive behaviour). Three additional mixed scales (social problems, thought problems and attention problems) do not belong to either domain. The Total Problems scale (TotP) represents overall impairment and is calculated as the sum of all syndrome scales plus 17 “other problems” items. In this study, we focused on the empirically derived CBCL composite domains (internalizing, externalizing, and total problems), which represent validated higher-order constructs widely used in epidemiological research. This approach was chosen to reduce multiple testing burden, improve interpretability, and ensure comparability with previous and future studies. The scores for each composite domain were treated as continuous variables, with higher scores indicating greater behavioural problems.

Cognitive abilities of children were assessed by trained psychologists using the Italian version of the WISC-IV. The WISC-IV is a standardised and well-validated measure of cognitive ability with strong reliability and validity in both clinical and population-based samples (Italian standardization: internal consistency α > 0.95 for FSIQ; test–retest reliability r = 0.86–0.92) [26]: details regarding the validation of the Italian version of the test are available elsewhere [27]. The WISC-IV evaluates different aspects of the child’s neurodevelopment providing different index scores which, when combined, yields the Full-Scale Intelligence Quotient (FSIQ) [28]. The FSIQ is the measure of the child’s overall cognitive ability and it is standardised to a mean of 100 and a standard deviation of 15, based on reference data from the Italian population, with scores ranging from 40 to 160. Higher FSIQ values represent better performance and, consequently, stronger cognitive abilities.

### 2.6. Exposures

PA performed by children at 7 years of age was assessed through the Supplementary Questionnaire completed by the parents (predominantly mothers, ~85%; ~15% were completed by fathers). Respondents were not provided with formal physiological definitions (e.g., MET intensity thresholds); instead, items relied on plain-language descriptions and concrete device examples. Specifically, PA was assessed using item Q50, which asked parents to report the usual number of days per week in which the child engaged in at least 1 h of physical activity (sports or active play, with explicit instructions to exclude physical activity undertaken at school), with the explicit instruction to exclude physical activity undertaken at school), with responses ranging from 0 to 7 days per week. The question referred to the child’s usual activity pattern and assessed the frequency of days with at least 1 h of activity, rather than total daily duration or intensity. The questionnaire did not include a separate assessment of the setting in which the activity occurred or a procedure for objectively verifying activities occurring outside parental observation, such as at school or with peers. However, a separate question in the same questionnaire assessed participation in extracurricular activities and specifically included sports as a response option. In our sample, approximately 89% of children participating in extracurricular activities were reported as participating in sports.

Taking a broader perspective of child movement within the local urban environment, “active play”, as reported by parents, predominantly represents structured or supervised activities. Given the specific topographical and urban context, a 7-year-old child’s ability to move around independently is limited, meaning that most PA outside the home likely requires parental organization and transportation. Interactive ST exposure was assessed via item Q54 as the parent-reported average number of hours per day during the school year that the child spent playing with video games, computer, tablets. Passive ST assessed as the parent-reported average number of hours per day during the school year that the child spent watching television. The exact wording of the parent-reported questions (Q50, Q54, and Q55) is provided in Supplementary Material S1 (Supplementary Parental Questionnaire).

### 2.7. Derivation of PA and Screen-Time Variables

Responses to the PA question were converted into the corresponding number of days per week, ranging from 0 to 7 days/week. Missing PA responses (*n* = 3, 0.6%) were handled via complete-case (listwise) deletion without imputation. For screen-time exposure (items Q54 and Q55), parents were asked to report the average number of hours per day their child spent watching television or using interactive screens during the school period, separately for weekdays and for weekends and holidays. The categorical responses were converted into estimated hours/day as follows: 0 h = 0, <1 h = 0.5, 1 h = 1, 2 h = 2, 3 h = 3, and 4 h = 4. For the open-ended category of ≥5 h/day, a value of 5.5 h/day was assigned as a representative value (lower boundary of 5.0 h plus a 0.5 h midpoint increment), representing standard epidemiological coding for top-coded continuous conversions to assign a plausible average for heavy screen users while avoiding extreme leverage points in linear models. Weekday and weekend/holiday estimates were then combined into a single average daily measure using weights of 4.8 and 2.2 days/week, respectively. The average weekly values of 4.8 school days and 2.2 non-school days were calculated by applying the statutory minimum requirement (200 teaching days) to the total duration of the school calendar period (approximately 290 calendar days between September and June, equivalent to roughly 41.5 weeks). Dividing 200 teaching days by 41.5 weeks yields an average of 4.8 school days per week across the school calendar period. The remaining 2.2 days per week represent non-school days (weekends, winter/spring term breaks, and public holidays).

These weights represent the average weekly distribution of weekdays and weekend/holiday days over the school-year period, accounting for school holidays rather than assuming a simple 5-day versus 2-day weekly distribution. Thus, the final estimate was calculated as [(weekday hours/day × 4.8) + (weekend/holiday hours/day × 2.2)]/7. The same procedure was applied separately to interactive screen use and television viewing. Responses coded as 999 were treated as missing; consequently, the derived screen-time variable was considered missing when either the weekday or weekend/holiday response was unavailable.

### 2.8. Potential Predictors and Confounders

Based on a literature review [29,30,31,32], the following potential predictors or confounding variables were identified and included in the study. During pregnancy: maternal IQ. At 1 month after delivery: child’s sex, household size as a socioeconomic indicator and family socioeconomic status (SES) index adapted from Bennett [33]. The SES index considered parental occupation (1 = yes vs. 0 = no), type of occupation (1 = white-collar vs. 0 = blue-collar, following Hollingshead classification), education level (0 = up to middle school, 0.5 = high school, 1 = university degree) and home ownership (1 = yes)—scores were summed, with occupation type and education averaged across parents. At the age of 7 years: child’s body mass index (BMI) z-score, calculated as weight (kg) divided by height squared (m^2^) and converted into WHO age- and sex-standardised z-scores [34] and exposure to secondhand smoke indoors (yes/no).

### 2.9. Statistical Analysis

The main characteristics of mother–child pairs were described by mean, standard deviation (SD) and median in the case of continuous variables and by frequency and percentage in the case of categorical variables. Socio-demographic and lifestyle information was collected from gestation to age 7 years. Multiple linear regression models were used to examine the associations of PA and ST with CBCL domains—internalizing problems (IntP), externalizing problems (ExtP), and total problems (TotP)—as well as with WISC-IV-derived FSIQ. The linear PA term was included as part of the primary analysis. To explore potential non-linear associations between PA and the outcomes, a quadratic term for the centered PA variable was additionally included in the regression models. The quadratic PA analyses were not preregistered and were therefore considered exploratory. For ST, only linear terms were included. PA was centered prior to generating the quadratic term to reduce collinearity between the linear and quadratic terms and improve the stability and interpretability of the regression coefficients. When evidence of a U-shaped relationship was observed, the vertex of the fitted quadratic curve was estimated using the following formula:

(1) $$X_{\mathrm{vertex}}=-\frac{B_{\mathrm{linear}}}{2B_{\mathrm{quadratic}}}$$

where B_linear_ and B_quadratic_ are the regression coefficients of the linear and quadratic PA terms, respectively. Because centered PA terms were used in the regression models, the estimated vertex was subsequently back-transformed to the original PA scale by adding the mean value of PA. In the final multiple regression models, a backward stepwise selection procedure based on a *p*-value threshold of ≤0.15 was applied to reduce model complexity while retaining potentially relevant predictors. The threshold of 0.15 was used as a predictor-retention criterion and was not used to define statistical significance, which was assessed using the conventional two-sided *p* < 0.05 threshold. No formal adjustment for multiple comparison was applied. Given the exploratory nature of the quadratic analyses, these results were considered hypothesis-generating and were interpreted using effect estimates, 95% confidence intervals, and two-sided *p*-values < 0.05. Statistical analyses stratified by child’s sex were also conducted. As a sensitivity analysis, the regression models were additionally fitted retaining all prespecified covariates, irrespective of their *p*-values, to assess the robustness of the findings to the variable-selection procedure.

The level of PA and child’s sex were forced to remain in the final model. Adjusted beta coefficients (B) and the 95% confidence interval (95%CI) were estimated. Given the potential interplay between exposures, we additionally examined whether the association between PA and each CBCL domain varied according to different levels of ST exposure. For the FSIQ score, we similarly explored whether the association between PA and FSIQ differed according to z-score BMI levels: these interaction analyses were considered exploratory and were not part of the primary study hypotheses. The assumptions of the linear regression models were assessed for each fitted model by visual inspection of residual-versus-fitted plots to evaluate linearity and homoscedasticity, and normal Q–Q plots of standardized residuals to assess residual normality. The Breusch–Pagan test was also performed to assess heteroskedasticity. All multivariable linear regression models were conducted using complete-case (listwise) deletion restricted to the variables included in each specific model; no missing data imputation was performed. In the primary multivariable stepwise regression models, dropping candidate predictors that did not meet the retention threshold maximized statistical power by retaining all available complete cases for the variables remaining in each final model. STATA (StataCorp. 2025. Stata Statistical Software: Release 19. College Station, TX, USA: StataCorp LLC) was used for the statistical analysis.

## 3. Results

A total of 900 pregnant women were initially enrolled in the cohort, of whom 767 (85%) remained in the study at delivery. At age 7 years, a total of 482 children (representing 62.8% of the cohort remaining at delivery and 53.6% of the initially enrolled prenatal cohort) were still participating. The mothers of children lost to follow-up at 7 years had a lower IQ (median 120 vs. 125, *p* < 0.001), and their families had a lower SES index (median 2.5 vs. 2.8, *p* = 0.007) compared with those of children who were followed up; on the other hand, the size of the homes in which they lived was similar to that of the families who were followed up. The main characteristics of the 482 mother–child pairs are presented in Table 1. Overall, according to parental reports, 12.5% of their children engaged in PA on ≤1 day/week. Remarkably, 4.6% did not do any PA, and only 2.1% conducted PA for at least 60 min across all seven days of the week. Weekly PA engagement did not differ between sexes (Mann–Whitney U *p* = 0.103). Among the 482 mother–child pairs evaluated at age 7 years, missing data for individual lifestyle, environmental, and outcome variables were minimal: physical activity (*n* = 3, 0.6%), passive screen time (*n* = 3, 0.6%), passive smoke exposure (*n* = 3, 0.6%), home size (*n* = 4, 0.8%), Family Socioeconomic Index (*n* = 6, 1.2%), CBCL outcomes (*n* = 1 for each domain, 0.2%), and WISC-IV FSIQ (*n* = 6, 1.2%). Maternal IQ and child sex had no missing data (*n* = 0). Missingness was higher for child BMI z-score (*n* = 44, 9.1%) and interactive ST (*n* = 73, 15.1%), as deriving interactive ST required complete responses across both weekday and weekend/holiday items. No missing data imputation was performed; all regression analyses used complete-case (listwise) deletion restricted to the variables included in each model. In the primary stepwise regression models (Table 2), dropping non-retained candidate predictors maximized available complete cases (*n* = 472 for IntP, *n* = 423 for ExtP, and *n* = 428 for TotP). Complete data across all prespecified variables simultaneously were available for 360 children (*n* = 359 for WISC-IV FSIQ).

The results of multiple linear regressions, between each domain (IntP, ExtP and TotP) of CBCL and PA are presented in Table 2. There was evidence that the association between PA and IntP was better described by a quadratic function than by a simple linear function. The exploratory quadratic model indicated a positive association between the quadratic PA term and IntP. In the regression model, the quadratic PA term was directly associated with IntP (B_PA^2_ = 0.32, 95% CI 0.03; 0.61), whereas the linear PA term was inversely associated with IntP (B_PA_ = −0.77, 95% CI −1.54; −0.09). The fitted quadratic association was consistent with a U-shaped pattern, with IntP scores initially decreasing with increasing PA and then increasing at higher PA levels (estimated vertex ≈ 3.8 days/week).

**Figure 1:**
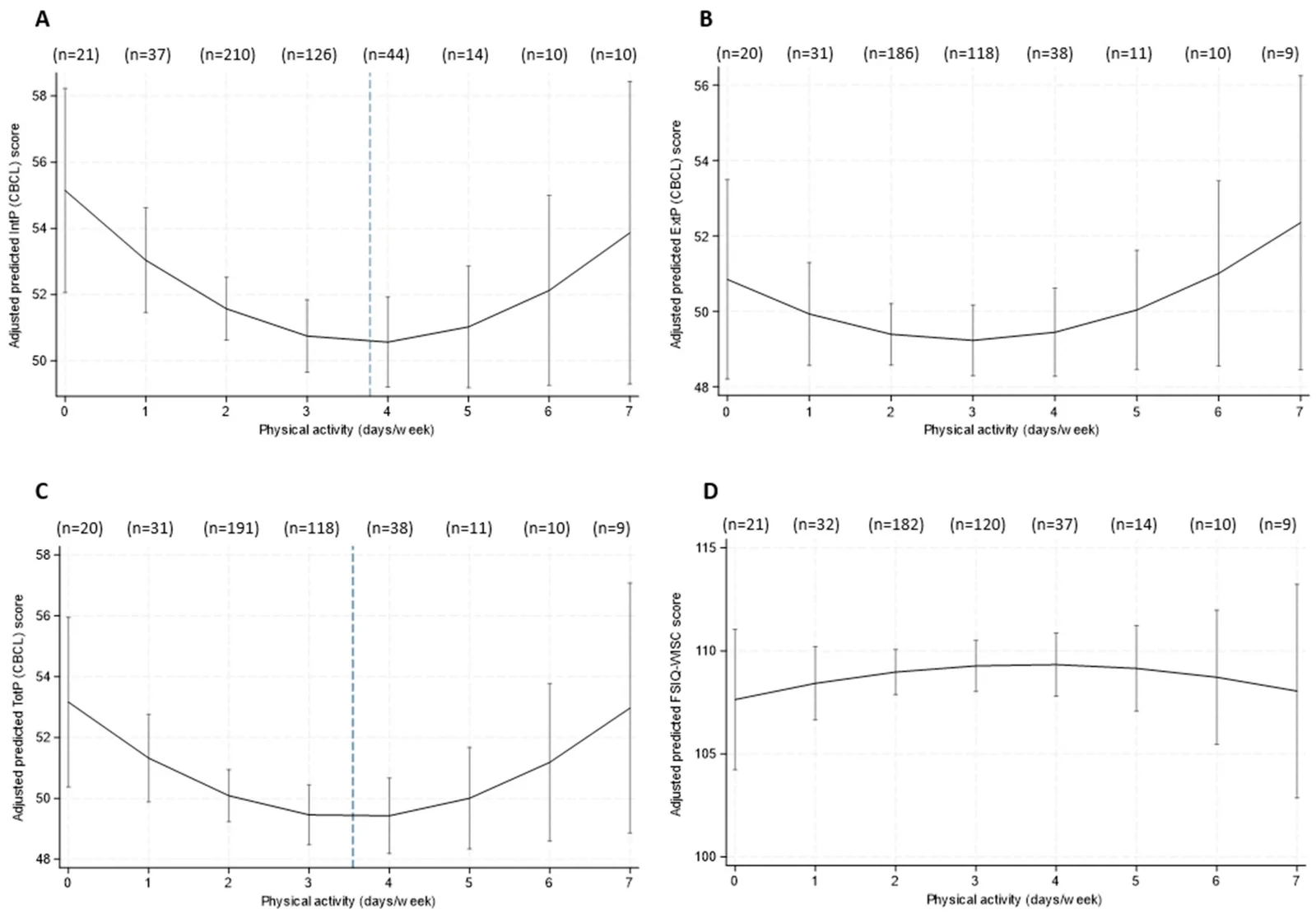
(**A**) Adjusted predicted Internalizing Problems (IntP) scores from the Child Behavior Checklist (CBCL) across physical activity levels. The exploratory quadratic model fit displays a non-linear pattern, with a model-estimated predicted-score minimum (vertex) occurring at approximately 3.8 days/week (vertical dashed line). (**B**) Adjusted predicted Externalizing Problems (ExtP) scores from the Child Behavior Checklist (CBCL) across physical activity levels. No statistically significant linear or quadratic association was observed. (**C**) Adjusted predicted Total Problems (TotP) scores from the Child Behavior Checklist (CBCL) across physical activity levels. The exploratory quadratic model fit displays a non-linear pattern, with a model-estimated predicted-score minimum (vertex) occurring at approximately 3.5 days/week (vertical dashed line). (**D**) Adjusted predicted Full-Scale Intelligence Quotient (FSIQ) scores from the Wechsler Intelligence Scale for Children (WISC-IV) across physical activity levels. No statistically significant linear or quadratic association was observed.

This pattern is illustrated in Figure 1A (adjusted model-based marginal effects). For ExtP there was no evidence of an association with PA level nor of a non-linear pattern of either a linear or a quadratic association between PA and ExtP (Table 2, Figure 1B).

Similarly, for TotP, the association with PA was better described by a quadratic function. The quadratic PA term was positively associated with TotP (B_PA^2_ = 0.30, 95% CI 0.04 to 0.56), and the fitted quadratic association was consistent with a U-shaped pattern. The estimated vertex of the fitted curve was approximately 3.5 days/week of PA (Figure 1C), indicating that the lowest predicted TotP scores occurred around 3.5 days/week of PA, with higher scores observed at lower and higher activity frequencies.

Passive ST and passive smoke exposure were strongly associated with higher CBCL scores across domains, while higher BMI z-scores were associated with increased ExtP and TotP and, overall, girls had lower problems than boys.

No evidence of interaction effects was detected between PA and passive ST in any CBCL domain.

In the multivariable linear regression analysis examining FSIQ, there was no evidence of either a linear or a quadratic association between PA and FSIQ (linear PA term: B_PA_ = 0.28, 95% CI −0.57; 1.14; quadratic PA term: B_PA^2_ = −0.12, 95% CI −0.44; 0.20) (Table 3, Figure 1D). Maternal cognitive ability was positively associated with children’s FSIQ (B = 0.25, 95% CI 0.15; 0.35), as was family socioeconomic status (B = 1.48, 95% CI 0.34; 2.62). Higher BMI z-scores were inversely associated with FSIQ (B = −0.96, 95% CI −1.88; −0.06). No evidence of an interaction between PA and BMI z-score was observed.

Plots reflect adjusted model-based predictions derived from the primary stepwise regression models (Table 2 and Table 3). Numbers above activity categories indicate category sample sizes (*n*). Curves represent exploratory parametric quadratic model fits rather than raw observed outcome trends. Note that the quadratic analyses are exploratory, and the highest activity categories contain relatively few participants.

All fitted linear regression models satisfied the underlying model assumptions based on graphical diagnostics and the Breusch–Pagan test, with no evidence of heteroskedasticity (*p* > 0.5).

In addition, the potential effect-modifying role of sex on the associations between the exposure variables and study outcomes was explored through sex-stratified analyses and formally assessed by including interaction terms in the multivariable models. These interaction terms did not improve model fit, and no evidence of effect modification by sex was observed.

Full multivariable regression models, including all potential predictors, are reported in Supplementary Tables S1 and S2. In sensitivity analyses retaining all prespecified covariates, the overall direction of associations and estimated vertex locations for non-linear PA terms were consistent with the primary stepwise models (approx. 3.8 days/week for IntP and approx. 3.4 days/week for TotP). However, the statistical evidence for certain associations—particularly for IntP—was less statistically robust in these fully adjusted specifications. Specifically, while point estimates and trajectory shapes remained similar, the quadratic PA term for internalizing problems (B = 0.30, 95%CI −0.02; 0.62, *p* = 0.063) and passive ST (B = 1.34, 95%CI −0.23; 2.70, *p* = 0.054) crossed the conventional significance threshold, as did the quadratic PA term for TotP (B = 0.25, 95%CI −0.02; 0.52, *p* = 0.064). In contrast, passive ST remained positively associated with CBCL ExtP (B = 1.30, 95%CI 0.20; 2.41, *p* = 0.021) and TotP (B = 1.61, 95%CI 0.46; 2.77, *p* = 0.006). Interactive ST estimates were not statistically significant across any outcome in either the primary or full prespecified models, although the confidence intervals indicate uncertainty around the estimates; these findings suggest that the lack of statistical significance was not an artifact of backward stepwise variable selection. Complete model estimates for all prespecified variables are provided in Supplementary Tables S1 and S2. ExtP (B_PA^2_ = 0.16, 95%CI −0.10; 0.42) and FSIQ (B_PA^2_ = −0.09, 95%CI −0.45; 0.27) spanned zero.

## 4. Discussion

We analyzed the relationship between PA and mid-childhood behavioural and cognitive outcomes in an Italian birth cohort that was previously evaluated for chemical and alcohol exposures in early-life [35,36,37]. Overall, parents reported relatively low frequency of PA among the children, with only 2.1% of children reported to engage in at least one hour of sports or active play on all seven days of the week. These results partially align with findings from a 2023 Italian national survey, which reported that 19.2% of children never engage in PA and 11.1% do so no more than one day, while 39.3% are active two days, 27.8% three to four days, and only 2.5% participate in sports 5–7 days per week [38]. We believe that the relatively low frequency of reported PA may partly reflect limited opportunities for free, unorganized play within the local urban environment. The specific built environment and topographical features of the area may constrain safe, accessible spaces for spontaneous outdoor play near residential areas, thereby increasing reliance on organized or family-assisted physical activities.

The exploratory quadratic models suggested that children engaging in PA approximately 3–4 days per week had the lowest predicted internalizing and total problem scores. This general pattern is broadly compatible with previous observational evidence linking PA with more favourable emotional and behavioural outcomes in children, including studies using multivariable regression-based approaches, although the literature is heterogeneous and findings vary according to how PA is defined, measured, and modelled [1,2,39]. However, the fitted quadratic association suggests that the inverse association with behavioural problems does not continue linearly at higher levels of activity. Accordingly, the estimated vertex and the ascending portion of the fitted quadratic curve should be interpreted with caution, as they are based on relatively sparse data at the highest PA levels (e.g., 0–1 and 5–7 days/week), which may result in less stable estimates and greater uncertainty. In addition, residual confounding cannot be excluded. While some unstructured PA may occur during weekends or holidays, these opportunities are heavily dependent on family lifestyle and resources. To account for this, we included the SES as a core covariate in our multivariable models. This allows us to adjust for the potential confounding effect of socioeconomic status on both the access to organized sports and the likelihood of engaging in family-led outdoor activities during non-school days. We found no evidence of either a linear or a quadratic association between PA and ExtP, suggesting that these behaviours may be less responsive to generic PA and may require more specific activity. The fitted quadratic association between PA and TotP appeared to be primarily driven by the corresponding association with internalizing problems.

At age 7 years, our findings show lower IntP and, although borderline, ExtP behaviours in girls compared with boys. This is partially consistent with the literature, which suggests that boys display higher levels of externalizing behaviour during childhood, particularly before the age of 10 [40,41,42] and a relative increase in internalizing behaviours in girls after the age of 10 [40,43,44].

We observed a strong cross-sectional association between passive ST exposure (e.g., television viewing) and increased IntP, ExtP and TotP, indicating higher reported behavioural problem scores among children with greater television exposure. Consistent with this, previous studies report that higher total ST is associated with increased internalizing symptoms and behavioural difficulties [45], with both passive and interactive ST contributing to adverse outcomes [32]. In contrast, in our findings, interactive screen exposure was not associated with a worsening of behavioural domains (*p* > 0.15). One possible explanation is that interactive ST use may involve greater cognitive and motor engagement (e.g., attention, decision-making, and goal-directed behaviour) compared with passive screen viewing, which is more consistent with receptive consumption. This interpretation is consistent with theoretical frameworks suggesting that interactive digital activities may place higher demands on executive functions than passive media exposure [46,47], potentially leading to differential associations with emotional and behavioural regulation. Additionally, passive screen exposure may occur for longer durations or at more sensitive times of day (e.g., evenings), potentially accounting for stronger observed associations with behavioural problem scores. In our population, mean daily passive ST exposure was 1.36 h (median = 1.31), whereas interactive ST exposure was substantially lower (mean = 0.67; median = 0.50 h). This shorter interactive ST exposure may have limited the possibility of detecting associations. However, these interpretations remain speculative and should be confirmed in future studies.

Higher BMI z-scores were consistently associated with increased ExtP and, borderline, with TotP, suggesting an independent cross-sectional association between overweight status and behavioural problem scores. Previous studies have reported similar associations between childhood overweight and behavioural difficulties [48,49], particularly externalizing symptoms. The external consistency of our findings supports the validity of the observed associations between predictors and outcomes.

Passive smoke exposure was consistently associated with higher internalizing, externalizing, and total behavioural problems. Beyond possible neurotoxic effects, it may also reflect broader family environmental factors, including household stress, parenting practices, and socioeconomic disadvantage. Indoor smoking may reflect psychosocial stress or adverse family environments contributing to children’s behavioural difficulties, consistent with previous studies [31,50]. Sensitivity analyses incorporating the full set of prespecified covariates demonstrated consistent directionality and vertex alignment with our primary models, supporting a similar non-linear trajectory for PA (Supplementary Tables S1 and S2). However, these fully adjusted models revealed reduced statistical robustness for internalizing and total problems, where confidence intervals slightly crossed the null. Rather than viewing these findings as fully confirmed or definitive, they are best interpreted as showing a consistent non-linear pattern whose statistical precision is sensitive to full covariate control and sample retention.

For FSIQ, estimates for linear and quadratic PA terms as well as ST variables were not associated in either the primary or fully adjusted models. Although the wide confidence intervals indicate uncertainty around these estimates and preclude ruling out modest associations, overall findings suggest that global intelligence at age 7 years is more strongly linked to familial cognitive and socioeconomic factors. Furthermore, these results should not be interpreted as definitive evidence that no association exists, nor do they exclude potential PA benefits or adverse ST impacts on specific unassessed cognitive domains (such as executive functions or attention). No sex differences in FSIQ were observed, consistent with evidence showing comparable general intelligence and structural equivalence of the WISC across sexes [51,52]. Overweight was also inversely linked with the full-scale intelligence, consistent with prior findings of an inverse association with FSIQ scores. This finding aligns with prior research suggesting that higher body mass in childhood may be linked to lower global cognitive functioning. Several mechanisms may underlie this association. Biologically, excess adiposity is linked to low-grade systemic inflammation and neuroendocrine alterations affecting brain regions involved in executive function, attention, and memory [53]. Psychosocially, overweight children may experience stigma, social exclusion, or reduced engagement in cognitively stimulating activities, which may co-occur with lower cognitive test performance to identify preventive interventions that may improve behavioural health of school-age children [48].

### Limitations and Strengths

Our study has several limitations. Although the study is embedded within a prospective birth cohort, the exposures and outcomes examined in the present analyses were assessed concurrently at age 7 years. Thus, these findings should be interpreted as cross-sectional associations within a prospective cohort rather than as evidence of prospective developmental effects. PA and ST were not the primary exposure originally targeted in the cohort, and their impact on neurodevelopment was not part of the initial study hypothesis. However, the potential difficulty in distinguishing between structured sports and free play may be partially mitigated by the local urban context, where limited neighborhood play spaces mean that parent-reported PA largely reflects structured or organized activities. PA was collected using a semi-quantitative measure—frequency of days per week with at least one hour of activity—without detailed information on activity type, context, duration beyond one hour, or intensity. This limited level of detail may introduce exposure misclassification and limit our ability to distinguish between different forms of activity (e.g., structured sports vs. free play or individual sports vs. team sports). Similarly, although interactive and passive ST exposures were collected and analyzed separately, ST measures were based on broad parent-reported estimates of daily exposure and did not capture qualitative aspects such as content type, multitasking behaviours, context of use, or patterns of engagement across different devices. Such measurement constraints could lead to imprecise estimates of associations with behavioural and cognitive outcomes. Additionally, for the open-ended top category of screen time (≥ 5 h/day), a fixed value of 5.5 h/day was assigned as a standard coding assumption. Because alternative values for this open-ended upper category were not evaluated in formal sensitivity analyses, this specific coding assumption represents a potential measurement limitation. Furthermore, no specific reliability or validity assessment was available for the parent-reported PA and ST questions used in this study, as these questions were part of the Supplementary Parental Questionnaire rather than a separately validated exposure instrument. Consequently, no instrument-specific recommendations regarding their reliability, validity, or interpretation are available. Nevertheless, the PA question explicitly referred to “sport or active play” and asked parents to report the number of days per week their child was physically active for at least one hour, excluding school-based activity. In addition, a separate question on extracurricular activities (Supplementary Material S1—Supplementary Parental Questionnaire) included sports as a response option, and approximately 89% of children participating in extracurricular activities were reported to participate in sports. Although this does not constitute formal validation of the PA question, these findings provide some contextual support for interpreting the measure as reflecting sport and active play, while its lack of formal validation should be considered when interpreting the observed association. Moreover, PA assessment, as well as other covariates included in the final models, was based on self-reported questionnaires rather than objective measures (e.g., accelerometers for PA). This approach may introduce recall bias and potentially result in either over- or under-reporting of the true values [54,55]. Previous studies comparing parent-reported PA with accelerometry have shown moderate agreement and a tendency for parents to overestimate their children’s PA levels [56,57]. This potential measurement error should therefore be considered when interpreting the observed associations. Although reverse causation cannot be fully excluded, the concurrent assessment of exposures and outcomes at age 7 years limits our ability to establish the temporal direction of the observed associations. For example, children with greater behavioural difficulties may receive more television exposure, or parents may use television to manage challenging behaviours. However, the CBCL assesses behavioural symptom severity rather than functional or medical impairments that would directly limit PA or screen use. Elevated scores primarily reflect behavioural dysregulation rather than physical or health-related constraints, making it less likely that CBCL-defined problems alone would directly explain reduced PA. Similarly, WISC-derived FSIQ reflects global cognitive functioning; lower FSIQ scores do not necessarily indicate limitations that would directly preclude engagement in PA or ST. Nevertheless, behavioural and cognitive factors may indirectly influence activity patterns, and this possibility cannot be excluded. Therefore, the observed associations should not be interpreted as evidence of causal effects of PA or ST on behavioural or cognitive functioning. Finally, the NAC-II cohort may have limited generalizability to populations with different socioeconomic or environmental distributions.

This study also has several strengths. By assessing both behavioural functioning and cognitive performance at age 7 years, we were able to examine how children’s PA and ST habits at this pivotal age differentially relate to multiple developmental outcomes. Additionally, we assessed both behavioural and cognitive outcomes using standardised instruments, one of which (FSIQ) administered by two trained psychologists, whose evaluations showed a high level of inter-rater reliability [58]. Furthermore, our analyses for both behavioural and cognitive outcomes accounted for multiple potential confounders and related factors, allowing for a more accurate estimation of the independent association between PA and ST and developmental outcomes. Moreover, the relatively large sample size provided adequate precision for the main analyses, particularly given the young age of the study population.

## 5. Conclusions

Taken together, these findings suggest that regular PA in childhood, with the lowest predicted internalizing and total problem scores occurring at approximately 3–4 days per week, is associated with fewer behavioural problems, while its relationship with externalizing behaviours and global intelligence are weak. Internalizing, externalizing, and total behavioural problems occurred among children who spent more time watching television. Characteristics of PA and ST should be studied further to inform potential preventive strategies targeting child behavioural health.

## Figures and Tables

**Table 1:** Characteristics of 482 Italian children and their mothers from the NAC-II cohort at 7 years.

Child Sex, *n* (%):	
Male	252 (52.3)
Female	230 (47.7)
Child age (years), mean ± SD (median)	7.1 ± 0.1 (7.1)
Child height (cm), mean ± SD (median)	124.8 ± 5.3 (124.5)
Child weight (kg), mean ± SD (median)	26.4 ± 4.8 (25.5)
Child BMI z-score, mean ± SD (median)	0.7 ± 1.1 (0.6)
Child engagement in physical activity for at least one hour (times per week), mean ± SD (median)	2.3 ± 1.3 (2.0)
Frequency, *n* (%)	
Never	22 (4.6)
1	38 (7.9)
2	210 (43.6)
3	128 (26.6)
4	44 (9.1)
5	17 (3.5)
6	10 (2.1)
7	10 (2.1)
Not reported	3 (0.6)
Daily hours of interactive screen exposure *, mean ± SD (median)	0.67 ± 0.56 (0.5)
Daily hours of passive screen exposure **, mean ± SD (median)	1.36 ± 0.74 (1.3)
Child’s passive smoke exposure, *n* (%)	
No	440 (91.3)
Yes	39 (8.1)
Not reported	3 (0.61)
Maternal occupational status, *n* (%)	
Employed	404 (83.8)
Housewife	37 (7.7)
Seeking work	19 (3.9)
Student	7 (1.5)
Stopped working	7 (1.5)
Other/missing	8 (1.7)
Maternal IQ ^§^, mean ± SD (median)	119.3 ± 11.0 (125.0)
Family Socioeconomic Index, mean ± SD (median)	2.7 ± 0.9 (2.8)
HOME SIZE, *n* (%)	
<50 m^2^	38 (7.9)
50–100 m^2^	313 (64.9)
>100 m^2^	127 (26.4)
Not reported	4 (0.8)
Child Behavior Checklist—CBCL, mean ± SD (median)	
Internalizing Problems	51.6 ± 9.7 (50.0)
Externalizing Problems	49.4 ± 8.2 (50.0)
Total Problem	50.1 ± 8.6 (50.0)
Full-Scale IQ (FSIQ) WISC-IV ^†^, mean ± SD (median)	109.0 ± 11.0 (109.0)

* Child’s use of personal computer or gaming devices; ** child’s use of television; ^§^ Raven’s progressive matrices score; ^†^ Wechsler Intelligence Scale for Children-Fourth Edition.

**Table 2:** Associations of physical activity, screen-time exposure and potential confounders with behavioral problems tested with Children Behavior Check List (CBCL) among 482 children from the PHIME-NAC-II birth cohort at age 7 years: multiple linear regression analysis.

				CBCL					
	Internalizing Problems (*n* = 472)			Externalizing Problems (*n* = 423)			Total Problems (*n* = 428)		
	B	95% CI	*p*	B	95% CI	*p*	B	95% CI	*p*
Physical activity	−0.77	−1.54; −0.09	0.048	−0.13	−0.80; 0.53	0.691	−0.59	−1.29; 0.11	0.100
Physical activity ^2	0.32	0.03; 0.61	0.029	0.19	−0.06; 0.43	0.132	0.30	0.04; 0.56	0.022
Daily hours of passive screen time *	1.44	0.29; 2.60	0.014	1.49	0.50; 2.49	0.003	1.88	0.83; 2.92	0.000
Sex									
Male	Reference	-	-	Reference	-	-	Reference	-	-
Female	−2.28	−4.00; −0.57	0.009	−1.29	−2.77; 0.19	0.087	−1.08	−2.63; 0.48	0.176
Family Socioeconomic Index				−0.63	−1.43; 0.17	0.123			
Passive smoke exposure									
No	Reference	-	-	Reference	-	-	Reference	-	-
Yes	4.85	1.64; 8.05	0.003	2.86	0.07; 5.65	0.044	4.51	1.58; 7.45	0.003
Child BMI z-score				0.78	0.10; 1.46	0.024	0.70	−0.01; 1.41	0.054
	**R-Squared = 0.062**			**R-Squared = 0.075**			**R-Squared = 0.076**		

PA was mean-centered before creating the quadratic term. Therefore, the coefficient for the linear term represents the association between PA and the outcome at the mean PA level. Abbreviations: B = regression coefficient; CI = confidence interval; PA = physical activity (times per week for at least one hour/day engagement); * child’s use of television; BMI = Body Mass Index.

**Table 3:** Associations of physical activity and potential confounders with child’s general intellectual functioning measured by WISC-IV Full-Scale IQ (FSIQ) among 482 children from the PHIME-NAC-II birth cohort at age 7 years: multiple linear regression analysis.

		WISC-IV FSIQ	
	B	95% CI	*p*
Physical activity	0.28	−0.57; 1.14	0.513
Physical activity ^2	−0.12	−0.44; 0.20	0.458
Sex			
Male	0	--	--
Female	−0.16	−2.14; 1.82	0.876
Maternal IQ *	0.25	0.15; 0.35	0.000
Child BMI z-score	−0.96	−1.86; −0.06	0.037
Family Socioeconomic Index	1.48	0.34; 2.62	0.011
R-Squared = 0.115			

Physical activity was mean-centered before creating the quadratic term. Therefore, the coefficient for the linear term represents the association between PA and the outcome at the mean PA level. Abbreviations: B = unstandardized regression coefficient; CI = confidence interval; PA = physical activity (times per week for at least one hour/day engagement); * Raven’s progressive matrices score; BMI = Body Mass Index.

## Data Availability

The data presented in this study are available on request from the corresponding author. The data are not publicly available due to privacy and ethical reasons.

## Supplementary Materials

The following supporting information can be downloaded at: https://www.mdpi.com/article/10.3390/children13101442/s1, S1: Supplementary parental questionnaire at the 7-Year Follow-Up; Table S1: Sensitivity analyses of associations between physical activity, screen time, and CBCL scores using the full prespecified covariate set; Table S2: Sensitivity analyses of associations between physical activity, screen time, and WISC-IV FSIQ scores using the full prespecified covariate set.

## Abbreviations

The following abbreviations are used in this manuscript:

BMI	Body Mass Index
CBCL	Child Behaviour Checklist
ExtP	Externalizing Problems
FSIQ	Full-Scale Intelligence Quotient
IntP	Internalizing Problems
IQ	Intelligence Quotient
ISS	Istituto Superiore di Sanità (Italian National Institute of Health)
NAC-II	Northern Adriatic Cohort II
PA	Physical Activity
PHIME	Public Health Impact of Long-Term, Low-Level Mixed Element Exposure in Susceptible Population Strata
SD	Standard Deviation
SES	Socioeconomic Status
ST	Screen Time
TotP	Total Problems
WHO	World Health Organization
WISC	Wechsler Intelligence Scale for Children
WISC-IV	Wechsler Intelligence Scale for Children—Fourth Edition
