# Supplementary material for: Physical Activity, Screen Time, and Neurobehavioural Function at Age 7 Years: The Italian PHIME-NAC-II Cohort
Source: Children (Basel). 2026 Oct 21;13(10):1442. doi: 10.3390/children13101442 (PMC13702923; doi:10.3390/children13101442)
Supplement: Supplementary file 1 [file children-13-01442-s001.zip › Supplementary Tables S1 and S2.pdf]

Supplementary Table S1. Sensitivity analyses of associations between physical activity, screen time, and CBCL scores using the full prespecified covariate set.

	CBCL								
	**Internalizing Problems (N = 360)**			**Externalizing Problems (N = 360)**			**Total Problems (N = 360)**		
	**B**	**95% CI**	**p**	**B**	**95% CI**	**p**	**B**	**95% CI**	**p**
**Physical activity**	-0.89	-1.75; -0.03	0.043	-0.27	-0.96; 0.43	0.691	-0.56	-1.30; 0.17	0.131
**Physical activity ^2**	0.30	-0.02; 0.62	0.063	0.16	-0.10; 0.42	0.132	0.25	- 0.02; 0.52	0.064
**Daily hours of passive screen time***	1.34	-0.23; 2.70	0.054	1.30	0.20; 2.41	0.021	1.61	0.46; 2.77	0.006
**Daily hours of interactive screen time****	0.97	-0.92; 2.87	0.312	0.77	-0.77; 2.30	0.327	1.12	-0.48; 2.72	0.170
**Home size**									
Less than 50 m2	Reference	--	--	Reference	--	--	Reference	--	--
50-100 m2	1.47	-2.24; 5.17	0.437	-0.69	-3.69; 2.32	0.655	0.89	-2.24; 4.03	0.577
More than 100 m2	0.11	-3.93; 4.15	0.956	-0.91	-4.19; 2.37	0.586	0.46	-3.00; 3.88	0.793
**Sex**									
Male	Reference	--	--	Reference	--	--	Reference	--	--
Female	-2.39	-4.45; -0.33	0.023	-1.21	-2.87; 0.46	0.157	-0.78	-2.52; 0.96	0.378
**Family Socioeconomic Index**	0.20	-0.97; 1.36	0.740	-0.67	-1.61; 0.28	0.167	-0.33	-1.32: 0.65	0.505
**Passive smoke exposure**									
No	Reference	--	--	Reference	--	--	Reference	--	--
Yes	5.23	1.57; 8.92	0.005	2.76	-0.22; 5.74	0.069	4.28	1.18; 7.39	0.007
**Child BMI z- score**	0.39	-0.50; 1.29	0.390	0.96	0.23; 1.68	0.010	0.91	0.15; 1.67	0.019
**Maternal IQ ¥**	-0.05	-0.15; 0.05	0.331	0.04	-0.04; 0.11	0.383	-0.02	-0.11; 0.06	0.583
	**R-Squared= 0.098**			**R-Squared= 0.086**			**R-Squared= 0.085**		

Physical activity was mean-centered before creating the quadratic term. Abbreviations: B = regression coefficient; CI = confidence interval; PA = physical activity (times per week for at least one hour/day engagement); *Child's use of television; ** Child's use of personal computer or gaming devices; BMI = Body Mass Index; ¥ Raven's progressive matrices score; Estimates are from multivariable linear regression models including the full set of prespecified covariates, without backward stepwise selection. Analyses were restricted to complete cases for all variables included in the corresponding model (N=360 for each CBCL outcome). B, unstandardized regression coefficient; CI, confidence interval.

Supplementary Table S2. Sensitivity analyses of associations between physical activity, screen time, and WISC-IV FSIQ scores using the full prespecified covariate set.

	**WISC-IV FSIQ (N = 359)**		
	**B**	**95% CI**	**p**
**Physical activity**	0.50	-0.47; 1.47	0.312
**Physical activity ^2**	-0.09	-0.45; 0.27	0.638
**Daily hours of passive screen time***	-0.35	-1.89; 1.20	0.658
**Daily hours of interactive screen time***	-0.57	-2.71; 1.57	0.600
**Home size**			
Less than 50 m2	Reference	--	--
50-100 m2	-0.27	-4.44; 3.91	0.901
More than 100 m2	1.06	-3.50; 5.62	0.647
**Sex**			
Male	Reference	--	--
Female	-0.98	-3.31; 1.35	0.409
**Family Socioeconomic Index**	1.79	0.47; 3.11	0.008
**Passive smoke exposure**			
No	Reference	--	--
Yes	1.86	-2.23; 5.96	0.371
**Child BMI z- score**	-1.04	-2.06; -0.01	0.048
**Maternal IQ***	0.24	0.13; 0.34	0.000
	**R-Squared= 0.12**		

Physical activity was mean-centered before creating the quadratic term. Abbreviations: B = regression coefficient; CI = confidence interval; PA = physical activity (times per week for at least one hour/day engagement); *Child's use of television; ** Child's use of personal computer or gaming devices; BMI = Body Mass Index; ¥ Raven's progressive matrices score; Estimates are from multivariable linear regression models including the full set of prespecified covariates, without backward stepwise selection. Analyses were restricted to complete cases for all variables included in the corresponding model (N=359 for WISC-IV FSIQ outcome). B, unstandardized regression coefficient; CI, confidence interval.
