# Supplementary material for: Physical Activity, Screen Time, and Neurobehavioural Function at Age 7 Years: The Italian PHIME-NAC-II Cohort
Source: Children (Basel). 2026 Oct 21;13(10):1442. doi: 10.3390/children13101442 (PMC13702923; doi:10.3390/children13101442)
Supplement: Supplementary file 1 [file children-13-01442-s001.zip › Supplementary Material S1.pdf]

PHIME

FOLLOW-UP

SUPPLEMENTARY QUESTIONNAIRE

Child age: 7 years

EPIDEMIOLOGICAL STUDY ON THE HEALTH OF MOTHER AND NEWBORN

IRCCS Burlo Garofolo - Trieste
University of Udine - Chair of Hygiene and Epidemiology

QUESTIONNAIRE CODE	I_ I_ I_ I_ I_ I_ I*

Fields marked with an asterisk (*) are for study staff only and should not be completed by the respondent.

MOTHER - CONTACT DETAILS

Q1 Mother's surname and given name

____

Home address

Q2 Since completing the last questionnaire / attending the last study visit, have you changed your home address?

[ ] 1. Yes [ ] 2. No

If No, go to Q8.

Q3 Street / square

____

Q4 House number

____

Q5 Municipality

___________________________ Q5CODE /_/_/_/_/_/*

Q6 Postcode

____

Q7 Province

_______ Q7CODE /_/_/*

Mother's telephone number

Q8 Telephone number

____

MOTHER - MARITAL STATUS

Q9 Since completing the last questionnaire / attending the last study visit, has your marital status changed?

[ ] 1. Yes [ ] 2. No

If No, go to Q11.

Q10 What is your current marital status?

[ ] 1. Married / cohabiting [ ] 2. Widowed
[ ] 3. Separated / divorced [ ] 4. Single

MOTHER - EMPLOYMENT

Q11 Since completing the last questionnaire / attending the last study visit, has your employment status, occupation or employment sector changed?

[ ] 1. Yes [ ] 2. No

If No, go to Q15.

Q12 What is your current employment status?

[ ] 1. Employed - on maternity/other leave
[ ] 2. Employed - currently working
[ ] 3. Homemaker
[ ] 4. Looking for work
[ ] 5. Student
[ ] 6. Retired
[ ] 7. Other (please specify): ___________________

Q12CODE /_/_/_/_/

Q13 Please state your occupation

Q14 Please state your employment sector

HUSBAND / PARTNER - EMPLOYMENT

Q15 Since completing the last questionnaire / attending the last study visit, has your husband/partner changed occupation or employment sector?

[ ] 1. Yes [ ] 2. No

If No, go to Q19.

Q16 What is your husband/partner's current employment status?

[ ] 1. Employed - on paternity/other leave
[ ] 2. Employed - currently working
[ ] 3. Homemaker
[ ] 4. Looking for work
[ ] 5. Student
[ ] 6. Retired
[ ] 7. Other (please specify): ________________________________

*Q16CODE /_ /_ /_ /_ /**

Q17 Please state your husband/partner's occupation

Q18 Please state your husband/partner's employment sector

PARENTS - OCCUPATIONAL CATEGORY AND EMPLOYMENT SECTOR

Q19 Please indicate the occupational category that best describes your occupation and that of your husband/partner.

ISCO-88 major groups	Mother	Husband / partner
1. Legislators, senior officials and managers		
2. Professionals		
3. Technicians and associate professionals		
4. Clerks		
5. Service workers and shop and market sales workers		
6. Skilled agricultural and fishery workers		
7. Craft and related trades workers		
8. Plant and machine operators and assemblers		
9. Elementary occupations		
10. Armed forces		

Q13CODE /_ /_ /_ /_ /   Q17CODE /_ /_ /_ /_ /**

Q20 Please indicate the employment sector that best describes your occupation and that of your husband/partner.

ISIC Rev.4 sections	Mother	Husband / partner
1. Agriculture, forestry and fishing		
2. Mining and quarrying		
3. Manufacturing		
4. Electricity, gas, steam and air-conditioning supply		
5. Water supply; sewerage, waste management and remediation activities		
6. Construction		
7. Wholesale and retail trade		
8. Transportation and storage		
9. Accommodation and food service activities		
10. Information and communication		
11. Financial and insurance activities		
12. Real estate activities		
13. Professional, scientific and technical activities		
14. Administrative and support service activities		
15. Public administration and defence		
16. Education		
17. Human health and social work activities		
18. Arts, entertainment and recreation		
19. Other service activities		
20. Activities of households as employers / domestic services		
21. Activities of extraterritorial organisations and bodies		

Q14CODE /_ /_ /_ /_ /   Q18CODE /_ /_ /_ /_ /**

HOUSING CONDITIONS

Q21 Since completing the last questionnaire, have there been any changes in your housing conditions?

[ ] 1. Yes   [ ] 2. No

Q22 The home you currently live in is:

[ ] 1. Owner-occupied   [ ] 2. Rented
[ ] 3. Occupied free of charge / loan for use   [ ] 4. Living with parents / parents-in-law

Q23 How many bedrooms are there in your home?

____

Q24 What is the approximate floor area of your home?

[ ] 1. Less than 50 m²   [ ] 2. 50-100 m²   [ ] 3. More than 100 m²

Q25 How many people live in your home, including you and your child?

Adults (18 years or older) ____________
Children (under 18 years) ____________

Q26 How many cars does your household own?

(Enter 0 if none) ____________

Q27 What type of heating system do you have in your home?

[ ] 1. Fuel oil   [ ] 2. Natural gas (public supply)
[ ] 3. Coal   [ ] 4. Wood (stove)
[ ] 5. Diesel oil   [ ] 6. Other: ______________________________

CHILD - IDENTIFYING INFORMATION

Q28 Child's surname and given name

____

Q29 Date of birth

DD /_ /_ /   MM /_ /_ /   YYYY /_ /_ /_ /_ / _

Q30 Paediatrician's name

____

CHILDCARE

Q31 Who mainly looks after the child during the day from Monday to Friday? (More than one answer may be selected.)

[ ] 1. Mother   [ ] 2. Father
[ ] 3. Maternal grandparents   [ ] 4. Paternal grandparents
[ ] 5. Primary school   [ ] 6. Babysitter
[ ] 7. Other (please specify):

____

Q32 Who mainly looks after the child during the day at weekends? (More than one answer may be selected.)

[ ] 1. Mother   [ ] 2. Father
[ ] 3. Maternal grandparents   [ ] 4. Paternal grandparents
[ ] 5. Babysitter   [ ] 6. Other (please specify):

____

DIET

Q33 How often does your child eat the following types of fish or seafood? Please mark one frequency for each row; also indicate whether the usual amount is a half portion.

Type of fish / seafood, preparation and portion	Never	<1/month	1-3/month	1/week	2-4/week	5-6/week	1/day	2-3/day	>3/day	Half portion
Fish - boiled, grilled or baked (150 g)	1	2	3	4	5	6	7	8	9	Yes
Crustaceans (lobster, prawns, scampi, etc.) - boiled, grilled or baked (150 g)	1	2	3	4	5	6	7	8	9	Yes
Molluscs (octopus, squid, clams, mussels, etc.) - boiled, grilled or baked (150 g)	1	2	3	4	5	6	7	8	9	Yes
Fried fish (150 g)	1	2	3	4	5	6	7	8	9	Yes
Crustaceans (lobster, prawns, scampi, etc.) - fried (150 g)	1	2	3	4	5	6	7	8	9	Yes
Molluscs (octopus, squid, mussels, etc.) - fried (150 g)	1	2	3	4	5	6	7	8	9	Yes
Tuna, mackerel or sardines in oil (1 can, 80 g)	1	2	3	4	5	6	7	8	9	Yes

Mark the number corresponding to the selected frequency.

PARENTS - LIFESTYLE

Q34 Do you currently smoke?

[ ] 1. Yes
[ ] 2. No

If No, go to Q36.

Q35 How many cigarettes do you usually smoke per day?

1. Number of cigarettes ___________

[ ] 2. I do not smoke every day

Q36 Does your partner currently smoke?

[ ] 1. Yes
[ ] 2. No

If No, go to Q38.

Q37 How many cigarettes does your partner usually smoke per day?

1. Number of cigarettes ___________

[ ] 2. He/she does not smoke every day

Q38 On a usual day, how long does your child spend indoors with people (including you and your partner) who smoke in the child's presence?

[ ] 1. Never
[ ] 2. Less than 10 minutes/day
[ ] 3. 10-29 minutes/day
[ ] 4. 30 minutes to 1 hour/day
[ ] 5. 1 hour/day
[ ] 6. 2 hours/day
[ ] 7. 3 hours/day or more

Q39 Do you currently drink alcoholic beverages?

[ ] 1. Yes
[ ] 2. No
[ ] 3. Yes, but only on special occasions (birthdays, anniversaries, celebrations, etc.)

If No, go to Q41.

Q40 On average, how many units of alcoholic beverages do you drink per week? One unit = one glass of wine, one can of beer, or one small glass of spirits.

____

Example: 2 glasses of wine + 3 cans of beer + 1 small glass of spirits = 6 units/week.

Q41 Does your partner currently drink alcoholic beverages?

[ ] 1. Yes
[ ] 2. No
[ ] 3. Yes, but only on special occasions (birthdays, anniversaries, celebrations, etc.)

If No, go to Q43.

Q42 On average, how many units of alcoholic beverages does your partner drink per week? One unit = one glass of wine, one can of beer, or one small glass of spirits.

____

Example: 2 glasses of wine + 3 cans of beer + 1 small glass of spirits = 6 units/week.

CHILD HEALTH

Q43 Since completing the last questionnaire, has your child been admitted to hospital?

[ ] 1. Yes [ ] 2. No

If Yes, please provide details for each hospital admission:

Age	Reason for admission	Ward and hospital	Treatment

Q44 Excluding hospital admissions, has your child received any treatment?

[ ] 1. Yes [ ] 2. No

If Yes, please provide details for each episode:

Problem / condition	Treatment

Q45 Is your child currently taking any medication?

[ ] 1. Yes [ ] 2. No

If Yes, please specify:

Brand name	Reason	Times per day

CHILD HEALTH - MEDICAL DIAGNOSES

Q46 Has a doctor ever diagnosed your child with any of the following?

Condition	Diagnosis	If Yes
Asthma	[ ] Yes [ ] No	If Yes, number of episodes: ______
Asthmatic bronchitis or bronchospasm	[ ] Yes [ ] No	If Yes, number of episodes: ______
Bronchiolitis	[ ] Yes [ ] No	If Yes, number of episodes: ______
Bronchitis	[ ] Yes [ ] No	If Yes, number of episodes: ______
Bronchopneumonia or pneumonia	[ ] Yes [ ] No	If Yes, number of episodes: ______
Laryngitis, laryngospasm or croup	[ ] Yes [ ] No	If Yes, number of episodes: ______
Otitis	[ ] Yes [ ] No	If Yes, number of episodes: ______
Eczema or atopic dermatitis	[ ] Yes [ ] No	If Yes, number of episodes: ______

Q47 From birth to the present, has your child been diagnosed with a chronic disease or medical condition (including congenital malformations)?

[ ] 1. Yes [ ] 2. No

If Yes, please state the diagnosis: ____

Q48 Has a doctor diagnosed your child with any of the following?

Condition	Yes / No	If Yes, please specify
Food allergies	[ ] Yes [ ] No	
Food intolerances	[ ] Yes [ ] No	
Other allergies	[ ] Yes [ ] No	

CHILD - LIFESTYLE

Q49 Does your child take part in any extracurricular activities?

[ ] 1. Yes [ ] 2. No

If Yes, which activities? (More than one answer may be selected.)

[ ] 1. Sports
[ ] 2. Music and/or singing Hours/week: /_ /_ /_ /
[ ] 3. Language courses Hours/week: /_ /_ /_ /
[ ] 4. Other (please specify): ____________________ Hours/week: /_ /_ /_ /

Q50 During the school term, on average how many days per week is your child physically active (sport or active play) for at least one hour? Do not include physical activity undertaken at school.

[ ] 1. Never [ ] 2. 1 day [ ] 3. 2 days [ ] 4. 3 days
[ ] 5. 4 days [ ] 6. 5 days [ ] 7. 6 days [ ] 8. 7 days

Q51 Does your child regularly use digital/electronic devices (computer, tablet, mobile phone or smartphone)?

[ ] 1. Yes [ ] 2. No

Q52 If Yes, which devices? (More than one answer may be selected.)

[ ] 1. Computer [ ] 2. Tablet
[ ] 3. Smartphone [ ] 4. Other (please specify): ______________________________

Q53 For what purposes? (More than one answer may be selected.)

[ ] 1. Gaming [ ] 2. School-related activities
[ ] 3. Access to social media (WhatsApp, Twitter, Facebook, etc.) [ ] 4. Other (please specify): ______________________________

CHILD - SCREEN TIME

Q54 During the school term, on average how many hours per day does your child play video games (PlayStation, PSP, Wii, Nintendo DS, etc.) or use a computer, tablet, mobile phone or smartphone? Consider the morning before school, after school and the evening.

Weekdays

[ ] 1. Does not play/use these devices
[ ] 2. Less than 1 hour/day
[ ] 3. 1 hour/day
[ ] 4. 2 hours/day
[ ] 5. 3 hours/day
[ ] 6. 4 hours/day
[ ] 7. 5 hours/day or more

Weekends and public holidays

[ ] 1. Does not play/use these devices
[ ] 2. Less than 1 hour/day
[ ] 3. 1 hour/day
[ ] 4. 2 hours/day
[ ] 5. 3 hours/day
[ ] 6. 4 hours/day
[ ] 7. 5 hours/day or more

Q55 During the school term, on average how many hours per day does your child watch television (cartoons, films, TV series, DVDs, etc.)? Consider the morning before school, after school and the evening.

Weekdays

[ ] 1. Does not watch television
[ ] 2. Less than 1 hour/day
[ ] 3. 1 hour/day
[ ] 4. 2 hours/day
[ ] 5. 3 hours/day
[ ] 6. 4 hours/day
[ ] 7. 5 hours/day or more

Weekends and public holidays

[ ] 1. Does not watch television
[ ] 2. Less than 1 hour/day
[ ] 3. 1 hour/day
[ ] 4. 2 hours/day
[ ] 5. 3 hours/day
[ ] 6. 4 hours/day
[ ] 7. 5 hours/day or more

Q56 When your child watches television or uses digital/electronic devices, is he/she usually also engaged in other activities? (More than one answer may be selected.)

[ ] 1. No
[ ] 2. Yes - eating
[ ] 3. Yes - playing
[ ] 4. Yes - other activities (please specify): ______________________________

Q57 Does your child have a television in his/her bedroom?

[ ] 1. Yes
[ ] 2. No

Q58 Is there a Wi-Fi network in your home?

[ ] 1. Yes
[ ] 2. No

COMPLETION DETAILS

Date completed: DD /_ /_ / MM /_ /_ / YYYY /_ /_ /_ /_ /

Questionnaire completed by:

[ ] 1. Mother
[ ] 2. Father
[ ] 3. Another family member (please specify): ______________________________

Thank you for your time and cooperation. *- The PHIME research team*
